# Combining expert knowledge and machine-learning to classify herd types in livestock systems

**DOI:** 10.1038/s41598-021-82373-3

**Published:** 2021-02-04

**Authors:** Jonas Brock, Martin Lange, Jamie A. Tratalos, Simon J. More, David A. Graham, Maria Guelbenzu-Gonzalo, Hans-Hermann Thulke

**Affiliations:** 1 Department of Ecological Modelling, PG Ecological Epidemiology, Helmholtz Centre for Environmental Research GmbH-UFZ, Leipzig, Germany; 2 Animal Health Ireland, Carrick-on-Shannon, Co., Leitrim, Ireland; 3 Centre for Veterinary Epidemiology and Risk Analysis, UCD School of Veterinary Medicine, University College Dublin, Dublin, D04 W6F6 Ireland

**Keywords:** Computational biology and bioinformatics, Classification and taxonomy, Data mining

## Abstract

A detailed understanding of herd types is needed for animal disease control and surveillance activities, to inform epidemiological study design and interpretation, and to guide effective policy decision-making. In this paper, we present a new approach to classify herd types in livestock systems by combining expert knowledge and a machine-learning algorithm called self-organising-maps (SOMs). This approach is applied to the cattle sector in Ireland, where a detailed understanding of herd types can assist with on-going discussions on control and surveillance for endemic cattle diseases. To our knowledge, this is the first time that the SOM algorithm has been used to differentiate livestock systems. In compliance with European Union (EU) requirements, relevant data in the Irish livestock register includes the birth, movements and disposal of each individual bovine, and also the sex and breed of each bovine and its dam. In total, 17 herd types were identified in Ireland using 9 variables. We provide a data-driven classification tree using decisions derived from the Irish livestock registration data. Because of the visual capabilities of the SOM algorithm, the interpretation of results is relatively straightforward and we believe our approach, with adaptation, can be used to classify herd type in any other livestock system.

## Introduction

Herd production type can influence the behaviour of infectious diseases in livestock populations. Depending on the nature of the pathogen under study, the risk factors for introduction and spread, the route(s) of exposure, and the speed of spread can each vary considerably between different herd types (e.g.^1–4^). A herd type can be considered as a population of individual farms that have broadly similar enterprise and management patterns^5,6^. A detailed understanding of herd types is needed for animal disease control and surveillance activities, to inform epidemiological study design and interpretation and to guide effective policy- and decision-making.

A number of different methods have been used to classify herd types, including expert-based and statistical approaches^7^. Expert-based methods rely on the formulation of classification rules using a participatory approach. An example was presented by^8^ who classified the French cattle sector by applying assignment rules that had been developed following discussion with a wide range of stakeholders. For statistical approaches, clustering and dimensionality-reduction methods such as Principal Component Analysis (PCA) are by far the most frequently applied techniques (e.g.^9,10^). The main objective of the PCA approach is to reduce a usually large number of input variables into a set of synthetic variables (principal components), which are then used to form clusters. The use of this approach, and an understanding of its results, may require a level of technical and mathematical understanding which is not matched by all the participants, many of whom may have been chosen primarily for their knowledge in livestock husbandry. Logically, it is helpful to use a method which combines the advantages of both approaches, including expert participation and the formal identification of important data dimensions, as well as outputs that are understandable by all participants.

Self-organising maps (SOMs), also known as Kohonen maps, are a machine-learning algorithm that projects high-dimensional input data into topology-preserving maps. These maps represent the distribution of the input data records according to their similarity. Thereby, clusters and patterns in complex datasets can be visualized (e.g.^11–13^). The algorithm was first proposed by^14^ and has since been applied as an exploratory and visualization tool for complex datasets in a variety of fields, ranging from industry and finance to medicine and the natural sciences^15^. As the method does not transform the data into a new data space, unlike PCA, the interpretation of results is relatively straightforward. This provides the opportunity for expert knowledge to be incorporated during the exploration of multi-dimensional data.

Our paper proposes a new method for the classification of herd types in livestock systems by combining expert knowledge and SOMs. In this approach, SOMs are used as a visualization tool that allows multidimensional data to be viewed in a manner that is simple and easy to understand. We apply this approach to the cattle sector in Ireland where there is a need for a detailed understanding of herd types that can be adapted to different epidemiological questions, including on-going discussions on control and surveillance for endemic cattle diseases such as bovine viral diarrhoea (BVD) and infectious bovine rhinotracheitis (IBR). In this paper, we present our approach and discuss its benefits and transferability to other regions and livestock systems. Finally, we provide a decision tree for cattle herd classification in Ireland that can be used by stakeholders and decision makers to support planning in the context of animal-health decision-making.

## Data and methods

### Datasets

Data for this analysis was obtained from the Animal Identification and Movement (AIM) database maintained by the Department of Agriculture, Food and the Marine (DAFM) in Ireland. In accordance with EU requirements, the AIM database comprises records on all births, movements and disposals (i.e. origin, destination and date), tracking each individual bovine in Ireland from birth or import to death or export^16^. This information is recorded at an individual animal level, and each animal has its own unique identity (ID) or tag number, so that its sex, breed and birth date, as well as the IDs of its herd and dam, can be ascertained. Data is collected from farmers, markets (known locally as marts), abattoirs and licensed meat and live export locations.

Taking into account the patterns of calving in Ireland outlined by^17^, we accessed the database for three dates in 2017 (1st January, 1st May and 1st September) and extracted relevant demographic data for all cattle registered on these dates. Movement data was extracted for each day in 2017 (1st January–31st December 2017).

### Data processing

We classified all bovines in the dataset as being of beef or dairy breed type according to the detailed breed information provided in the AIM system. Based on the work of the Irish Cattle Breeding Federation (ICBF) and^17^, we classified animals as dairy if their dam and sire were of following breeds (i.e. not necessarily the same): Ayrshire, Brown Swiss, Holstein/Friesian, Jersey, Normande and Norwegian Red. Animals were classified as beef if their dam and sire were of the following breeds: Angus, Aubrac, Blonde d’Aquitaine, Belgian Blue, Belted Galloway, Bazadais, Charolais, Dexter, Galloway, Hereford, Highland, Irish Maol/Droimeann, Kerry, Longhorn, Limousin, Marchigiana, Montbeliarde, MRI/MRY, Piedmontese, Partenaise, Rotbunte, Romagnola, Salers, Shorthorn, Simmental. Cattle were classified as cross-bred if bred by a beef sire and a dairy dam or vice versa, based on the dairy and beef breeds listed here.

We then aggregated the individual animal data to herd level, initially classifying the resulting ~ 100,000 Irish cattle herds as being of beef, dairy, mixed or unknown. Following recommendations of ICBF, this was done by first considering herds to be breeding enterprises if the proportion of animals in a herd that had ever calved by September 1st 2017 (pCalvedAnimals) was ≥ 25%. These herds were then classified as being of dairy type if ≥ 70% of their animals were dairy according to the above explanation. In cases where this value was < 30%, herds were classified as beef enterprises. All other breeding herds were classified as mixed. Herds classified as non-breeding using the ICBF criterion were not further categorised (i.e. herd type unknown).

### Definition of second order variables

Table 1 lists second order variables on the herd level that were derived during the rule extraction process as described in section “*Rule extraction from self-organising-maps*”. The variables reflect relevant processes for livestock management, i.e. demographic parameters and transport statistics.

**Table 1:** Second order variables.

Group	Variable	Description	Calculation	Rationale
Demographic variables	pFemaleAnimals	Proportion of females in a herd in May, relative to herd size	$\frac{n Females May}{herd Size May}$	To get an overview of the sex distribution
	pDairyBreed	Proportion of dairy breeds in a herd in May, relative to herd size	$\frac{n Dairy Breed May}{herd Size May}$	To get an overview of the breed distribution
	pCrossBreed	Proportion of dairy-beef cross breeds in a herd in May, relative to herd size	$\frac{n Cross Breed May}{herd Size May}$	To extract pure breed herds
	pCalvedAnimals	Proportion of animals in a herd that have ever calved by September, relative to herd size	$\frac{n Calved Animals September}{herd ize September}$	To differentiate breeding and non-breeding herds
	pMalesBetween1&2Years	Proportion of males between 1 and 2 years of age in a herd in May, relative to all animals between 1 and 2 years of age	$\frac{n Males Between 1 And 2 May}{n Animals Between 1 And 2 May}$	To extract herds that keep their males for fattening
	pAnimalsLess30Days	Proportion of purchased animals in a herd that remain for less than 30 days before being sold, relative to all out moves in a year	$\frac{n Animals Less 30 Days}{n Out Moves}$	To extract dealer/trading herds
Transport variables	pOutMovesToSL	Proportion of animals in a herd sold to the slaughterhouse per year, relative to maximum herd size (maximum herd size = largest herd size during one of the three sampling dates)	$\frac{n Out Moves To Sl}{maximum Herd Size}$	To differentiate between herds that sell their animals directly to the slaughter and those that use dealer herds etc.
	pOutMovesToBirthHerd	Proportion of animals in a herd moved out which are returning to their birth herd, relative to all out moves (calculated over 1 year)	$\frac{n Out Moves To Birth Herd}{n Out Moves}$	To extract contract rearing herds
	pInMovesToBirthHerd	Proportion of animals in a herd moved in which are returning to their birth herd, relative to all in moves (calculated over 1 year)	$\frac{n In Moves To Birth Herd}{n In Moves}$	To extract non-rearing herds that use a contract rearer

### Self-organising-maps

An SOM is an unsupervised learning algorithm intended to project high-dimensional input data onto a low-dimensional map while preserving the topological properties of the input data^14^. It is a method for dimensionality reduction. The produced map (output layer) consists of a predefined grid of nodes that is iteratively trained according to the patterns of the input data^14,15^. Due to the structure of the algorithm, input vectors that are similar to each other will be represented by the same or a nearby node. This allows for a visual identification of clusters/classes in the output (a map). As a result, SOMs have the ability to visualize unclassified high-dimensional input data in a structured way.

**Figure 1:**
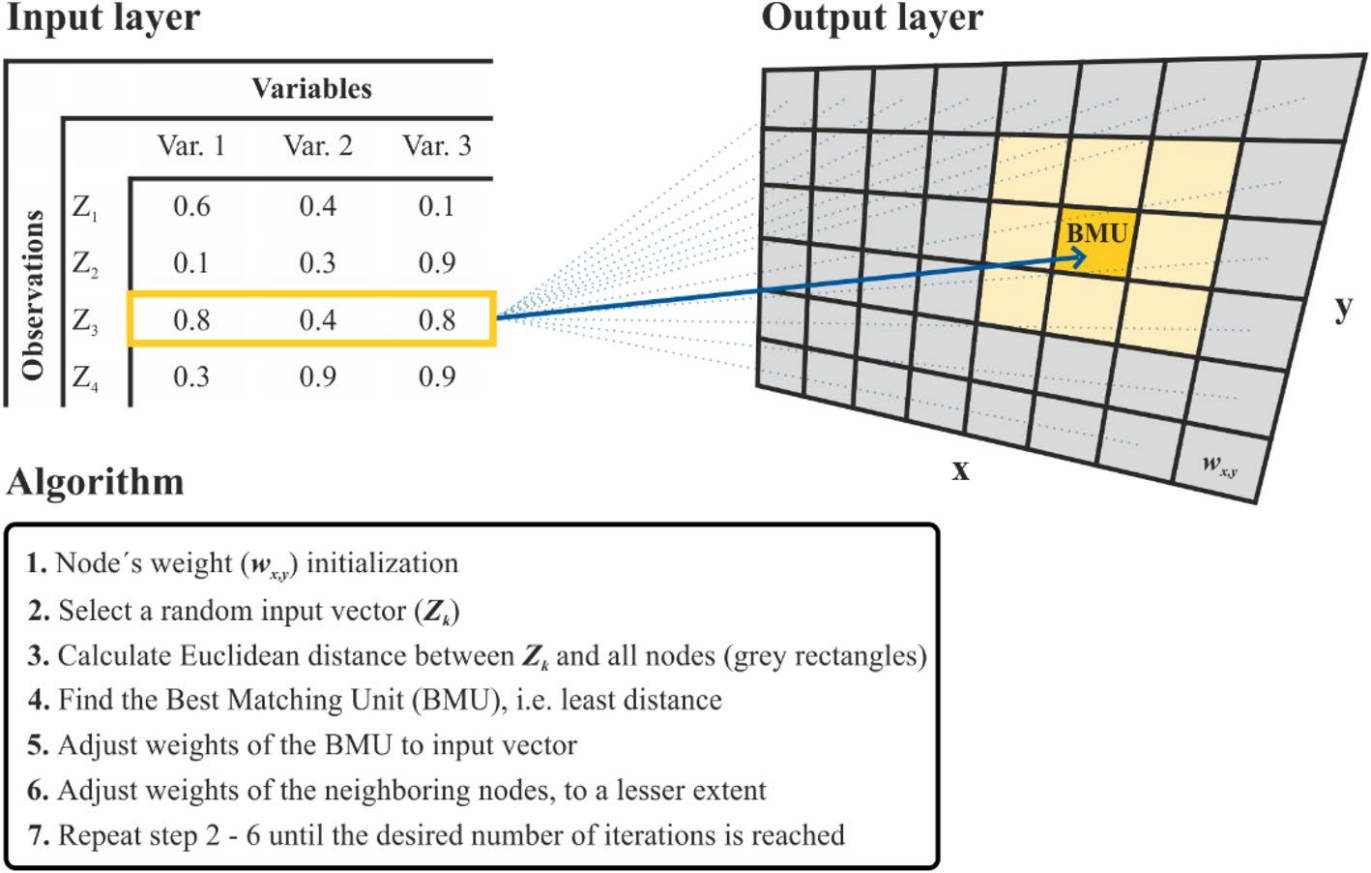
Architecture and learning process of a self-organising-map.

The functionality of the SOM is based on two layers, the input layer and the output layer (Fig. 1), and an iterative training procedure. The input layer are *K* vectors, each with N elements, formed by the (normalised) data records ***Z***_k_, k = 1,…, *K* and the variables Var 1 to Var N (Fig. 1). The output layer is represented by an (X, Y) matrix ***W.*** The matrix elements are weight vectors (***w***_***x,y***_) each with N elements, initialised with random values. During the iterative training process, the weight vectors are altered to best represent the vectors of the input layer. For each iteration, all data vectors are processed in random order. For each vector (***Z***_*k*_, marked by the yellow rectangle in the input layer), the most similar weight vector ***w***^*k*^_*x,y*_ of ***W*** is determined. Similarity is measured by the Euclidean distance between the input vector ***Z***_**k**_ and each weight vector ***w***_x,y_, and the weight vector with minimum distance is chosen:

1 $${{\varvec{w}}}_{x,y}^{k}=\underset{\forall x, \forall y}{\mathrm{min}}\left|{{\varvec{w}}}_{x,y}- {{\varvec{Z}}}_{k}\right|$$

The most similar vector is commonly called the Best Matching Unit (BMU in Fig. 1). After determining the BMU for the input vector ***Z***_k_, the values of corresponding weight vector ${{\varvec{w}}}_{{\varvec{x}},{\varvec{y}}}^{{\varvec{k}}}$ and all other weight vectors are adjusted. Adjustments depend on the distance between the position (x,y) of the weight vector (${{\varvec{w}}}_{{\varvec{x}},{\varvec{y}}}^{{\varvec{k}}}$) on the map and the position of the BMU, across the output layer. The adjustments are performed for all k (k = 1,…,K) in random sequence, according to:

2 $${{\varvec{w}}}_{x,y}={{\varvec{w}}}_{x,y}+ \boldsymbol{\alpha }\bullet {{\varvec{\beta}}}_{xy} \bullet \left({{\varvec{Z}}}_{k} - {{\varvec{w}}}_{x,y}\right),\mathrm{ \text{for all x and y}},$$

where $\boldsymbol{\alpha }$ is the learning rate, and ${{\varvec{\beta}}}_{xy}$ is a function which reduces adjustment with increasing distance to the BMU, e.g. a Gauss function. The adjustment of **W** is performed for all input vectors (i.e. ***Z***_k_ k in 1,..,***K***) in random sequence. For the subsequent iteration cycle (i.e. each input vector processed once), $\boldsymbol{\alpha }$ and the spread of function ${{\varvec{\beta}}}_{xy}$ are reduced.

As proposed by^18^, we applied a transformation to our data to normalise values per variable to mean 0 and variance 1. This was done to scale variables to compatible ranges and to reduce the effect of outliers in the data.

SOMs can be used for supervised classification problems by combing multiple output layers in different ways. For this purpose we used bi-directional Kohonen maps (BDKs) as described in^19^.

### Rule extraction from self-organising-maps

**Figure 2:**
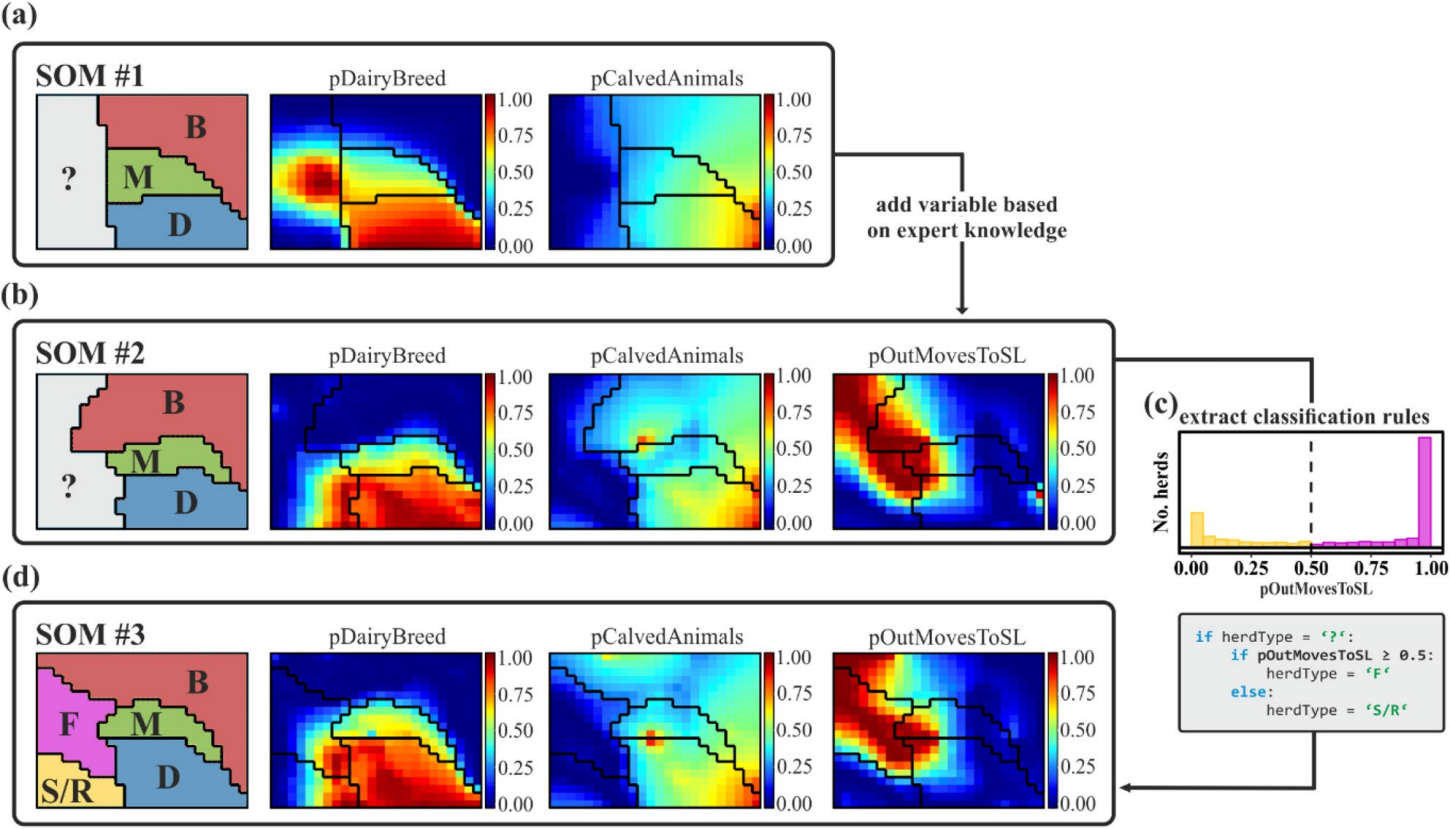
Rule extraction from self-organising-maps (SOMs). Class boundaries are represented as black lines and refer to beef (B), dairy (D), Mixed (M), fattening (F) and store/rearing (S/R) herds. The left most panel highlights the organisation of herd types in the SOM and the other panels show the component planes per input variable. Colour coding shows the values of the respective variable in a given pixel. (**a**) SOM #1 trained with 2 variables and pre-classified data (left panel). (**b**) SOM #2 trained with one additional variable (*pOutMovesToSL*) and its component planes. (**c**) Distribution of the *pOutMovesToSL* variable among the unclassified herds (grey region in left panel) and identification of a purposefully chosen threshold for class assignment i.e. 0.5. (**d**) SOM #3 trained with the 3 variables and the reclassified dataset (B, M, D plus newly F, S/R) and its component planes.

Extracting classification rules from a trained SOM can be divided into three main sub-tasks which are: (1) visual inspection of the structure of the SOM, (2) selecting attributes which would discriminate classes suggested by the SOM, and (3) deriving rules for assigning data records to these classes (see Fig. 2).

The output layer of an SOM comprises “component planes”, each representing the distribution of the values of a single input variable over the map. Component planes map the distribution of each variable onto the same geometry, i.e. following the last iteration update of the output layer. Plotting the component planes of an SOM allows the distribution of individual input variables to be inspected whilst simultaneously accounting for the similarity of input records across all variables.

To provide an example on how to read the component planes of the SOM and to extract classification rules, we have trained a supervised SOM (BDK) with our pre-classified data (i.e. by the ICBF rules) using two input variables, namely *pDairyBreed* and *pCalvedAnimals* (Fig. 2a). The left panel illustrates the areas in the corresponding map to which herds have been assigned by their pre-classification. The middle and right panel are the component planes of the two input variables, i.e. *pDairyBreed* and *pCalvedAnimals*. The colour code in the component planes refers to the value of the input variable in a given pixel of the map. Here we use red pixels to indicate high values of the respective variable, and blue for low values. We would like to stress that in all three panels a particular herd is always represented by the same pixel. Very similar herds are jointly represented by a single pixel. By looking at the component planes of the SOM #1, it is possible to recognise the pre-classification described in section “Data processing” in terms of the rule-forming variables (i.e. *pDairyBreed* and *pCalvedAnimals*). As an example, the unclassified non-breeding herds become clearly visible in the SOM output, jointly characterised by a minimal proportion of calved animals (i.e. blue area in right panel is coincident with the grey region in the left panel). Herds with a high proportion of animals of dairy breed (middle panel of Fig. 2a) are arranged at the bottom right to centre, i.e. the region where herds are represented that classify as Dairy or Mixed in the initial classification. However, the region of unclassified herds (grey in left panel of Fig. 2a) comprises blue as well as red areas in the *pDairyBreed* component plane (middle panel), i.e. herds with different proportions of dairy breed animals. This visual discrimination then leads to a search for useful sub-classifications.

To continue this illustrative example, the herds that are thus far not classified were subjected to further classification in subsequent steps. In this process, expert knowledge was introduced from a wide range of stakeholders [veterinarians from Technical Working Groups convened by Animal Health Ireland, analysts from ICBF, staff from Teagasc (the Agriculture and Food Development Authority) and researchers from UCD Centre for Veterinary Epidemiology and Risk Analysis (CVERA)]. From these experts we were told that in Ireland, non-breeding herds may be characterised by their practice to deliver animals for slaughter. Based on expert knowledge herds either sell their cattle to another herd prior to slaughter (we call these store or rearing-only herds; S/R) or send their cattle to the slaughter (fattening herds; F). To represent the difference, we added a third variable (*pOutMovesToSL*) to our input data and trained a new SOM based on this extended dataset and the initial classification (Fig. 2b). The examination of the additional component plane of the new SOM #2 reveals two sub-regions within the previously unclassified herds, namely herds with a high proportion of animals sent directly to slaughter and those with almost no direct moves to the slaughter. Ultimately this indicates that the newly introduced variable (*pOutMovesToSL*) is well suited as a means of separating fattening and store herds from each other.

The separability of previously unclassified non-breeding herds through the *pOutMovesToSL* variable can be visualized by a histogram (see Fig. 2c). The plot confirms the threshold for class assignment (here 0.5, i.e. 50%, direct moves to slaughter). Finally, a new supervised SOM (BDK) was trained on the dataset with the new classification, and checked to determine whether the class boundaries fit the distributions of the variables (see Fig. 2d).

The procedure of adding variables and identifying sub-classes was iteratively continued until the variety of cattle livestock management practices was covered. During this process, the introduction of expert knowledge has made it possible to find and test variables that can be used to distinguish herd types.

## Results

### Identified herd types

**Figure 3:**
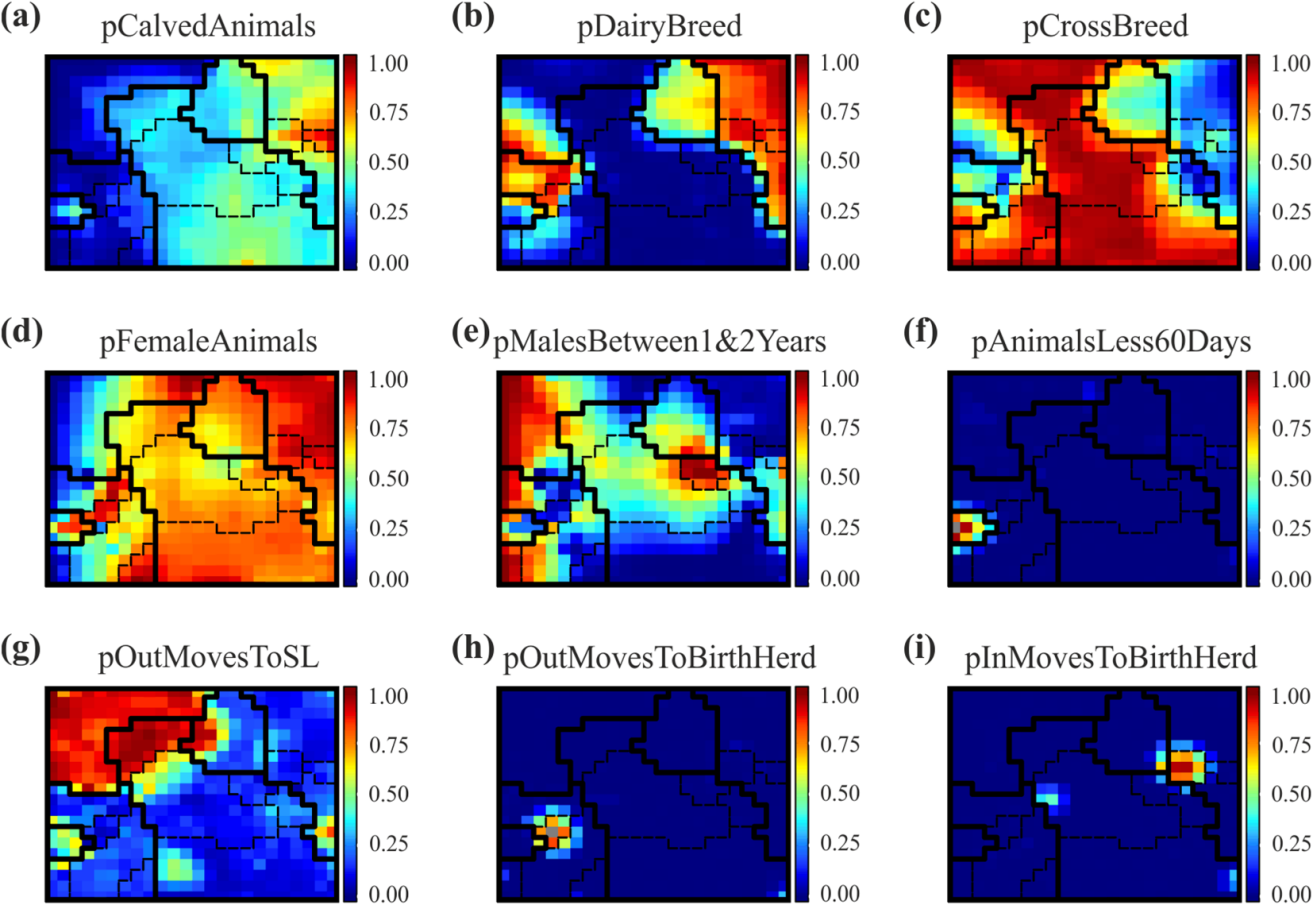
Component planes of the final self-organising map for the nine iteratively selected input variables, resulting in comprehensive discrimination of Irish cattle herds by 17 herd types (Fig. 4). Class boundaries for the six main herd types identified are represented as bold black lines, whereas sub-classes are separated by dashed lines. Herds are each represented by the same pixel in the nine panels. Colour coding shows the values of the respective variable in a given pixel.

**Figure 4:**
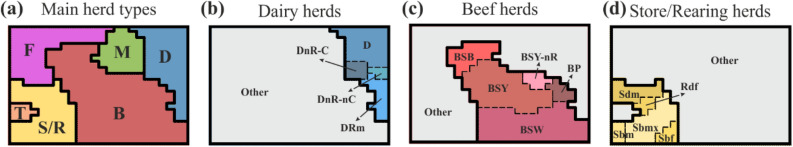
Class boundaries of the 17 identified herd types in the final self-organising map (see Fig. 3). (**a**) Main herd types in Ireland: dairy (D), beef (B), mixed (M), store/rearing (S/R), fattening (F), and trading (T) herds. (**b**) Dairy herd types: dairy (D), dairy no rearing—contract (DnR-C), dairy no rearing—no contract (DnR-nC), dairy rearing male calves (DRm). (**c**) Beef herd types: beef pedigree (BP), beef suckling to weanling (BSW), beef suckling to youngstock (BSY), Beef suckling to youngstock—no rearing (BSY-nR), Beef suckling to beef (BSB). (**d**) store/rearing herd types: store dairy males (Sdm), store beef males (Sbm), store beef females (Sbf), store beef mixed (Sbmx), rearing dairy females (Rdf).

The identification of relevant variables and extraction of herd types was performed as described in Methods, section “*Rule extraction from self-organising-maps*”. The component planes of the final SOM are presented in Fig. 3, including class boundaries. Bold lines indicate the six main herd types (see Fig. 4a): dairy herds (D), beef herds (B), mixed herds (M), store or rearing-only herds (S/R), trading herds (T) and fattening herds (F). Necessary sub-divisions are indicated by thinner dashed lines (see also Fig. 4b–d).

At the end of the process, each herd in the dataset is classified into one of 17 herd types. These herd types are explained in the following sections, with reference to Fig. 4.

#### Dairy herds

Among the dairy herds, we identified four sub-groups that differ in terms of their management practices (see Fig. 4b). Typical dairy herds (D) that sell their male calves at the age of a few weeks are the most prevalent dairy herd type. In these herds, most female calves are kept and reared as replacements. According to the component planes, these herds are characterized by a very high proportion of female animals (see Fig. 3d) and almost no males between the ages of 1 and 2 years (see Fig. 3e).

Non-rearing dairy herds (DnR-C) sell most of their calves, but female calves are moved to external contract rearing herds (Rdf, see section “Store/rearing herds”). Most of them return to the DnR-C herd as pregnant heifers before their first calving, which explains the increased values in the *pInMovesToBirthHerd* variable (see Fig. 3i). These herds mainly consist of cows. A similar herd type that was identified from the component planes is the DnR-nC herd type. There are similarities to the previous herd type, with the sale of most new-born calves within a few weeks after birth and the introduction of replacement heifers. In contrast to the DnR-C herds, however, replacements in DnR-nC herds are not returning from contract rearing herds, but from other herds with a surplus of cows or in-calf heifers. These herds have an above-average proportion of animals that have ever calved (see Fig. 3a). The component planes also reveal dairy herds with a high proportion of male animals between 1 and 2 years (see Fig. 3e). These herds were classified as dairy herds that also rear their male calves (DRm).

#### Beef herds (with breeding)

Five different types of (breeding) beef herds were identified from the data (see Fig. 4c). The first type we extracted is beef pedigree herds (BP), whose main production objective is breeding of purebred beef cattle. This herd type is characterized by a high proportion of purebred beef animals, i.e. a low share of both dairy (see Fig. 3b) and cross-bred (between dairy and beef) animals (see Fig. 3c). Beef pedigree herds are important for providing quality breeding stock to other commercial cattle producers in both the dairy and beef sectors.

The suckling to beef (BSB) herds follow the full beef production cycle, from birth through to the age of slaughter. Typically, calves stay with their dams until weaning at 6–8 months. Then, the weaned calves are retained for rearing and fattening in the same enterprise. Usually, these animals are sold to slaughter prior to two and a half years of age. Some females are kept as replacement heifers. This herd type can be distinguished by its high proportion of out moves to slaughter (see Fig. 3g).

The beef suckling to youngstock (BSY) system is very similar to the BSB system, however these herds do not fatten their cattle intended for slaughter. Instead, these animals are mainly sold to fattening herds as yearlings. This herd type becomes visible in the component planes by their equal proportions of male and female youngstock between 1 and 2 years (see Fig. 3e).

Non-rearing suckling to youngstock (BSY-nR) herds are a variation of the BSY herd type, with the difference being that most female calves are sold after weaning. Its main production objective is rearing of bulls and steers for beef production. These herds mainly purchase pregnant heifers for replacement from the market or other suckling herds. BSY-nR herds are characterized by a very high proportion of males between 1 and 2 years of age (see Fig. 3e).

Beef suckler to weanling (BSW) herds are the most common beef system in Ireland. These herds sell their male and some female calves after weaning between 6 and 8 months of age to store or fattening herds. A proportion of females are kept for replacement. These herds have almost no male youngstock aged between 1 and 2 years (see Fig. 3e).

#### Store/rearing herds

The store/rearing group only comprises herds that are non-calving. Overall, five sub-types were identified within this group, which differ in terms of the animals these herd preferentially rear (i.e. breed and sex) (see Fig. 4d). Store dairy male (Sdm) herds typically purchase young male calves from dairy herds. These animals are reared and then sold to fattening herds. This type of herd can be distinguished from others in the store/rearing group by its high proportion of dairy males (see Fig. 3d).

This study identified three different types of beef store herds, which differ in terms of their sex composition, rather than by management practices: store beef males (Sbm), store beef females (Sbf) and store beef mixed (Sbmx) systems (see Fig. 3d). These herds purchase beef animals as weanlings and rear them until they are sent to fattening herds.

In rearing dairy female (Rdf) herds, young female dairy calves are introduced, reared and inseminated, before being returned to their birth herd (DnR-C) as pregnant heifers. They become visible in the SOM output due to their increased proportion of out moves back to the birth herd (see Fig. 3h).

#### Mixed, trading and fattening herds

During analysis, we could identify herds which on average consist of half pure-bred dairy animals and half animals cross-bred between dairy and beef (see Fig. 3b,c). These herds produce milk on the one hand, but on the other hand they have another cattle enterprise, solely focused on beef production. We classified these dual purpose herds as mixed herds (M).

There are some non-breeding herds that have a high proportion of in and out moves and where the majority of animals remain in these herd for less than 30 days (see Fig. 3f). These herds are a kind of assembly point before animals are exported, sold to other herds or to the slaughterhouse. We classified them as trading herds (T).

Fattening herds (F) buy calves, weanlings, youngstock and cows from a wide range of herd types and fatten them until slaughter. Fattening herds do not normally produce their own calves, and the majority of their outward moves go to slaughter (see Fig. 3a,g). After considering the sex distribution within the fattening herds, it is noteworthy that it would be possible to continue to divide this type of herd into sex-specific fattening herds (see Fig. 3d). However, for many epidemiological problems this distinction is less important, as fattening can be considered a dead end for many pathogens because very few animals are sold from these herds to other herds, and usually no calvings occur.

### Extracted classification rules

**Figure 5:**
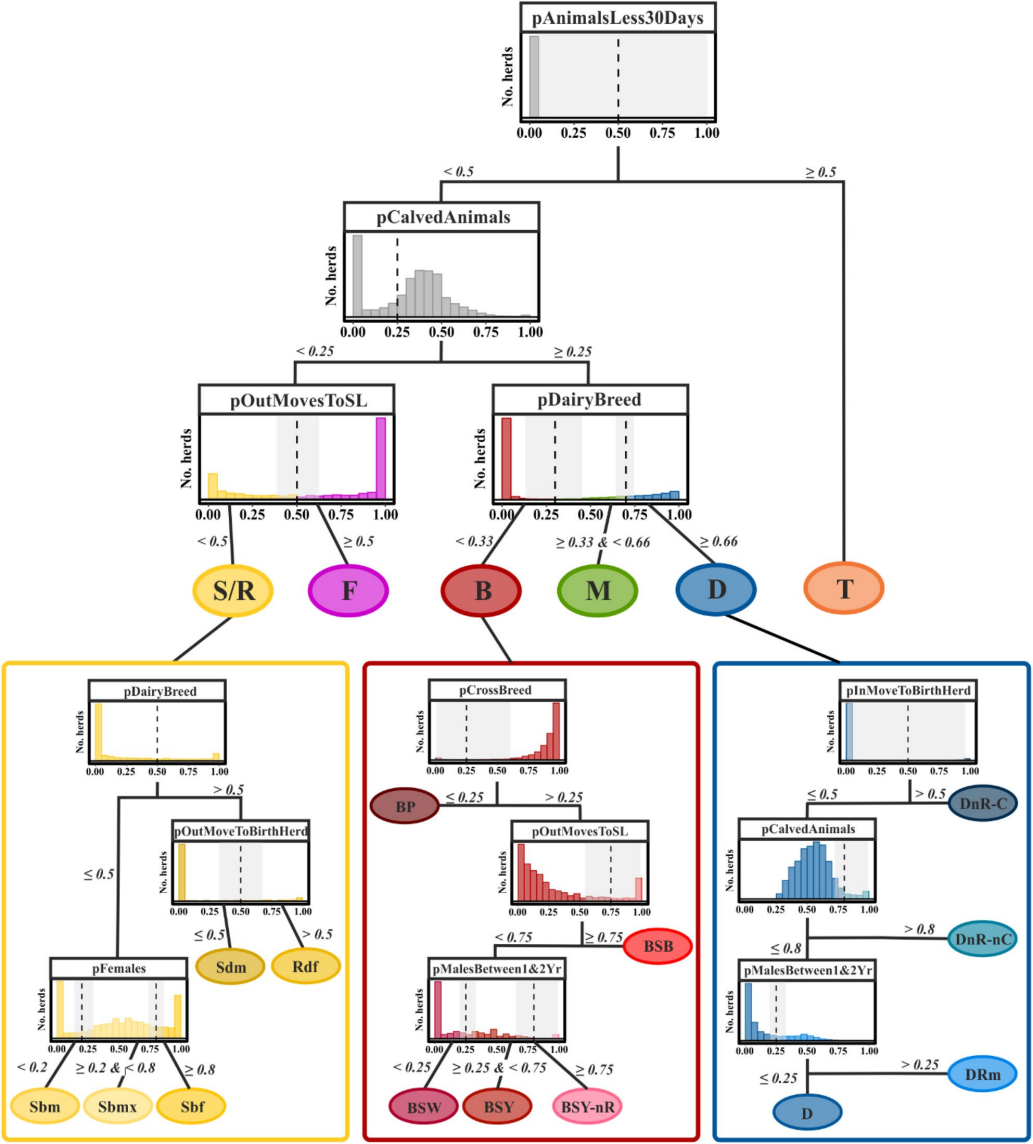
A decision tree for the classification of the Irish cattle sector. Histograms represent herds remaining at the respective node. Numbers and dashed line demarcate thresholds for class assignment. Main herd types: dairy (D), beef (B), mixed (M), store/rearing (S/R), fattening (F), and trading (T) herds. Dairy sub-types: dairy (D), dairy no rearing—contract (DnR-C), dairy no rearing—no contract (DnR-nC), dairy rearing male calves (DRm). Beef sub-types: beef pedigree (BP), beef suckling to weanlings (BSW), beef suckling to youngstock (BSY), beef suckling to youngstock—no rearing (BSY-nR), beef suckling to beef (BSB). Store/rearing sub-types: store dairy males (Sdm), store beef males (Sbm), Store beef females (Sbf), store beef mixed (Sbmx), rearing dairy females (Rdf). The grey rectangles among the thresholds indicate the range in which the classification threshold would have to be moved in order to assign 10% of the herds classified in the respective step to the other class.

Through examination of the component planes of the SOM, we developed a decision tree for the classification of the identified herd types (see Fig. 5). At each decision node, the distribution of the discriminating variable (see Table 1) of the *remaining* herds is presented as a histogram. Thresholds for class assignment (dashed lines) are given below the histogram. Overall, the classification comprises nine variables and 13 decisions. To classify a herd, a maximum of six of the 13 possible decisions are necessary.

## Discussion and conclusion

Livestock systems, including the Irish cattle sector, are often structured by production or breeding type. This information is important, noting that system classification can facilitate informed decision-making and protocol development. Due to the complexity and specialization of modern livestock management systems, however, such classifications rarely provide the details necessary for epidemiological risk analysis and modelling.

In this paper, we have combined expert knowledge and machine-learning methods to derive a sophisticated yet straightforward method to allow the Irish livestock system to be classified by characteristics derived from the AIM data, while reflecting herd management practices. To our knowledge, this is the first time that the SOM algorithm has been used to analyse and classify livestock systems. Using this approach, we have identified 17 herd types using 9 variables, applied in 13 decisions.

Herd type classification seeks to group herds into classes that each have broadly similar enterprise and management patterns. Trading networks, animal movements and age distributions derived from these classifications are more representative, providing valuable input for the development of targeted interventions or epidemiological models. As an example, herds assigned to the class non-rearing dairy herds (DnR-C) do not rear their own replacement heifers and sell most calves soon after birth. At the same time, these herds have to introduce in-calf heifers from a contract rearer for replacement of older dams. Hence, there will be herds that introduce calves, rearing them until they return to their birth herd as pregnant heifers. These were identified in this study as herds that rear dairy females (Rdf). Such refined data-driven structures capture regular and recurring dependencies between herds, which is very useful for risk assessment and contingency planning. A detailed understanding of such structural patterns will contribute to an improved understanding of disease epidemiology, including the eradication and surveillance of infectious diseases.

A recent example would be the new Animal Health Law (AHL) with its implementation principles that present new challenges for the management of livestock diseases^20^. Especially for the bovine sector, with the new EU regulations the herd management type became an important criterion for shaping the sampling strategy of national surveillance and control programs. With regard to BoHV-1 eradication as an example, the AHL prescribes certain rules on how herds can achieve a status of disease freedom. These rules often interact with specifics of the management regime in a herd. For example, the use of bulk tank milk testing alone can only be used in herds that have a minimum proportion of lactating cows. Due to normal replacement and breeding of offspring, a typical self-breeding dairy herd would never fall into this category. Non-rearing dairy herds (DnR_C and DnR_nC), on the other hand, could be checked by bulk tank milk testing alone. Such details become even more important when models are used to estimate program costs.Another recent example would be the planning of surveillance efforts in relation to the programme to eradicate bovine viral diarrhoea (BVD) from the Irish cattle population. The measures under discussion include the regular serological screening of youngstock in a herd as a test for undetected virus circulation. The approach is useful for dairy herds which raise their own replacements, but incompatible for herds in the dairy sector that rely on contract rearing.

Knowledge of some of the finer herd categories that we identified was already available through expert knowledge (e.g.^21^), however, explicit decision-support (as reflected in Fig. 5) was not formally available. This is reasonable, as a more complex stratification requires more detailed data management. However, through the combination of expert knowledge and the machine-learning procedure of SOMs, we could formally derive an improved means of categorisation. We now have a protocol at hand that is both logical and suitable for integration with the existing national cattle register.

The uncertainty associated with the classification thresholds that we have determined is part of the visual interpretation of the SOM, as the rule-extracting procedure is partially heuristic. The sensitivity of the classification thresholds can be understood from the histograms shown in Fig. 5, which illustrate the proportion of herds that would be assigned to a particular class depending on the potential change of the classification threshold. For some conditions, the threshold in the variable that determines the sub-classes becomes less robust the further down the decision tree is developed. However, the confirmation by expert knowledge input provides support in formally identifying less clear-cut thresholds based on defined characteristics of particular farm types, e.g. when subdividing beef store herds by their management and based on trading with particular sectors of the cattle population only (i.e. Fig. 5 left panel bottom decision). Interestingly, the SOM suggested a possible separation of herds based on the proportion of females. The experts were then able to provide reasoning in line with specific operations in the cattle sectors, but without naming the management principles explicitly.

According to^23^, the purpose for refinement of a herd categorisation should always be dependent on the problems that will be addressed. The classification we carried out sought to identify the general structures of cattle management processes in order to account for the explicit characteristics of identified herd types, e.g. during the planning of disease control/surveillance or in epidemiological models for cattle diseases. If the intended use was different (e.g. economic, environmental or sociological), it is certainly possible that other variables would be of interest and a modified classification could emerge when using the same SOM approach.

The proposed approach to combine expert knowledge with machine-learning is straightforward and can be adopted to any livestock system with comprehensive existing livestock registers. Several software packages for SOMs are publicly available and can be integrated in commonly used programming languages, such as R or MATLAB (e.g.^24–26^). The advantage of the SOM algorithm relates to the graphical representation, which can be directly interpreted together with livestock experts. Thus, the participatory procedure is facilitated by iterative cycles that include the addition of expert knowledge rules and graphical representation of the resulting structure.

## Data Availability

Data for this analysis was obtained from the Animal Identification and Movement (AIM) database maintained by the Department of Agriculture, Food and the Marine (DAFM) in Ireland. The data was made available for research purposes and cannot be made publicity available.
